# Beyond Immunosuppression: The Intricate Relationship Between Tacrolimus and Microangiopathy

**DOI:** 10.7759/cureus.49351

**Published:** 2023-11-24

**Authors:** Ripudaman S Munjal, Jagdish Sharma, Srinija Polishetti, Pushkar Sai Valleru, Himanshi Banker, Ramansh Bandhu Gupta, FNU Anamika, Rohit Jain

**Affiliations:** 1 Nephrology, Kaiser Permanente, Stockton, USA; 2 Internal Medicine, Manipal College of Medical Sciences, Pokhara, NPL; 3 Medicine, SVS Medical College, Hyderabad, IND; 4 Internal Medicine, Sri Devaraj Urs Medical College, Kolar, IND; 5 Medicine and Surgery, Maulana Azad Medical College, Delhi, IND; 6 Medicine, University College of Medical Sciences, Delhi, IND; 7 Internal Medicine, Penn State Health Hershey Medical Center, Hershey, USA

**Keywords:** chronic immunosuppression, drug induced, kidney transplant, thrombotic microangiopathy, tacrolimus

## Abstract

Tacrolimus, widely known as Prograf, has become the preferred immunosuppressant for preventing graft rejection in solid organ transplant recipients, particularly in steroid-sparing regimens. Its efficacy and reduced risk of acute and chronic rejection compared to cyclosporine have made it the preferred treatment option for transplant patients. However, tacrolimus has drawbacks as it is associated with adverse effects, such as renal tubular necrosis, kidney failure, hypertension, metabolic acidosis, and new-onset diabetes mellitus. Among the less common but potentially severe complications is thrombotic microangiopathy linked to tacrolimus usage. Identifying and addressing this condition early on is crucial given its severity and potential complications. Manifestations of this microangiopathy can vary, encompassing renal, neurological, cardiac, and respiratory symptoms, and, in some cases, presenting as pancreatitis, intestinal ischemia, or skin abnormalities. Although conventional management often involves plasma exchange as the primary therapeutic option, recent insights into the pathophysiology have led to newer drugs, such as eculizumab and belatacept, offering promising outcomes. In this narrative review, we delve deeper into the underlying pathophysiological mechanisms of tacrolimus-induced thrombotic microangiopathy and aim to provide clinicians with valuable recommendations for efficient and timely treatment strategies. By understanding the complexities of this condition and staying abreast of the latest advancements in therapeutic options, healthcare providers can optimize patient outcomes and ensure safer tacrolimus administration in solid organ transplant recipients.

## Introduction and background

Tacrolimus is a macrolide immunosuppressant first extracted from Streptomyces tsukubaensis in 1987 [1] and is used to prevent and treat graft rejection in organ transplant patients with other immunosuppressants. Tacrolimus can be given orally, sublingually, topical, or intravenously. Oral tacrolimus can be given in immediate-release (IR) or extended-release (ER) forms [2]. Usage of tacrolimus is limited due to its narrow therapeutic index and high inter- and intra-patient pharmacokinetic variability [3]. In the case of post-kidney transplantation, tacrolimus is started at low doses (0.5-1 mg twice daily), and, during the first week, the target level of tacrolimus is 6-9 ng/mL, followed by 5-8 ng/mL for one month; the dosage and goal tacrolimus levels can vary depending on the transplant center [4]. Tacrolimus can lead to nephrotoxicity and renal tubular necrosis and, in some individuals, exhibit metabolic acidosis and post-transplant diabetes mellitus, along with a range of adverse effects caused by immunosuppression [5]. The predominant gastrointestinal symptoms observed are abdominal pain, nausea, vomiting, and diarrhea and have also been associated with specific otic symptoms, such as tinnitus, otitis media, and otalgia [6]. Calcineurin inhibitors (CNIs) such as tacrolimus can cause cardiac hypertrophy, hypertension, dyslipidemia, and vascular remodeling [7]. Usage of tacrolimus is contraindicated if the patient has hypersensitivity, as the solvent used in injection is polyoxyl 60 hydrogenated castor oil (HCO-60) [6].

Thrombotic microangiopathy (TMA) can present as a complex array of conditions with diverse underlying causes; however, they share the standard pathophysiological features of microangiopathic hemolytic anemia, thrombocytopenia, and microthrombi formation, leading to ischemic tissue injury [8]. TMA includes congenital thrombotic thrombocytopenic purpura, hemolytic uremic syndrome, metabolism-mediated microangiopathy (genetic disorders of cobalamin), coagulation-mediated microangiopathy, drug-related TMA, stem cell transplant-related TMA, and pregnancy-related thrombotic syndromes (HELLP) [9]. In specific cases, TMA can occur during pregnancy, such as in cases of preeclampsia/eclampsia and HELLP syndrome. Certain drugs and toxins such as tacrolimus, quinidine, and ticlopidine can lead to drug-induced TMA. Another metabolic cause of TMA is cobalamin C deficiency [10].

Drug-induced TMA can arise through various mechanisms. For example, the use of quinine can cause the development of autoantibodies against platelet glycoprotein Ib/IX and IIb/IIa, leading to TMA. In another case, direct toxicity is observed with interferon β 82 [11]. It can be diagnosed based on microangiopathic hemolytic anemia, characterized by the observation of schistocytes in the peripheral smear, raised LDH levels, and thrombocytopenia [12]. CNIs (e.g., tacrolimus, cyclosporin) also cause dose-dependent toxicity, primarily affecting the kidneys [13]. Diagnosing drug-induced thrombotic microcytic angiopathy secondary to tacrolimus usage is essential, as discontinuing the drug serves diagnostic and therapeutic purposes [14]. Treatment includes the withdrawal of the causative drug (e.g., tacrolimus), and, in some cases, additional therapies such as plasmapheresis, usage of other immunosuppressants (e.g., eculizumab), and anticoagulation may be required [15].

In conclusion, our article sheds light on the critical issue of drug-induced TMA associated with tacrolimus usage. While highly effective in preventing graft rejection, Tacrolimus poses significant risks due to its narrow therapeutic index and high pharmacokinetic variability among patients. The development of TMA can result in severe complications, and its timely diagnosis is essential for graft survival and appropriate management. Our article highlights the importance of vigilance in monitoring patients on tacrolimus, considering the potential adverse reactions and their management to ensure successful organ transplantation outcomes.

## Review

Pathophysiology 

Tacrolimus is a potent immunosuppressant administered to transplant patients to reduce the risk of allograft rejection. Depending on the patient's condition, the drug can be given through various routes, such as oral, sublingual, topical, or intravenous. There are two oral variants: IR and ER. Tacrolimus has a variable half-life of inhibition, ranging from four to 41 hours to an average of 12 hours. It is primarily eliminated from the body through urine (2.4%) and bile routes (95%) [6]. However, using tacrolimus requires careful management due to its limited therapeutic index. Overexposure to the drug increases the risk of adverse effects, including nephrotoxicity, neurotoxicity, infections, malignancies, hypertension, diabetes, and gastrointestinal problems [6,7].

On the other hand, underexposure raises the likelihood of allograft rejection [16]. Tacrolimus targets T-cell activation; once it enters the lymphatic circulation, it inhibits calcineurin phosphatase, an essential enzyme in T-cell receptor signaling and cytokine production. This action helps protect allograft survival. The metabolization of tacrolimus in the liver and small intestine involves the cytochrome P450 3A5 (CYP35) enzyme. Additionally, tacrolimus acts as a substrate for the P-glycoprotein efflux transporter, which regulates its cellular distribution and intestinal absorption [17]. The drug's bioavailability varies from 5% to 90% among patients, with only 25% being available for its immunosuppressive effect. This limitation is due to 99% of tacrolimus binding to erythrocytes after entering the systemic circulation. Dissociated tacrolimus then exerts its immunosuppressive effect in the lymphatic system [18].

One of the potential adverse effects associated with using CNIs, such as tacrolimus, is TMA. Studies have reported that CNIs, including tacrolimus, can induce TMA in patients undergoing hematopoietic stem cell or solid organ transplantation [19]. TMA is characterized by end-organ damage caused by ischemia, affecting various circulatory systems. This can lead to kidney failure, neurological symptoms, cardiac complications, respiratory failure, vision impairment, pancreatitis, intestinal ischemia, and, occasionally, skin abnormalities [20].

Chatzikonstantinou et al. provided a comprehensive overview of the postulated mechanism of drug-induced TMA (DITMA) [21]. They proposed that the pathogenesis of DITMA involves several factors, including immune-mediated responses, dose-dependent or cumulative effects, ADAMTS13 deficiency, and complement activation [21] (summarized in Table 1). On the other hand, the exact pathogenesis of CNIs inducing TMA remains unclear. However, it is believed to result from increased thromboxane A2 production and decreased prostacyclin (PGI2) production. Additionally, direct damage to renal endothelial cells is implicated in causing thrombotic changes in small blood vessels, leading to eventual ischemia and damage to multiple organ systems [22,23]. Although the mechanism of direct endothelial injury is not fully understood, it is suggested to be either immune-mediated or dose-dependent direct toxicity [24]. This hypothesis is supported by the observation of the timing of TMA occurrence, the pattern of the disease, and the exclusion of other plausible explanations through thorough investigation. The suspicion of DITMA is further reinforced when TMA resolves upon withdrawal of the drug or when the recurrent endothelial injury occurs upon re-exposure to the drug [21].

**Table 1:** All the major diagnostic criteria for transplant-associated thrombotic microangiopathy (TA-TMA) Abbreviations: BMT-CTN (bone marrow transplant clinical trials network); IWG (international working group); MAHA (microangiopathic hemolytic anemia); LDH (lactate dehydrogenase); HPF (high-power field) 1. Thrombocytopenia - <50,000/μL or >50% reduction from baseline 2. Renal dysfunction - doubling of serum creatinine from the baseline or 50% reduction in creatinine clearance 3. Hypertension - blood pressure >95th percentile for ages less than 18 years and >140/90 mm Hg for ages more than 18 years 4. Proteinuria - random urine protein concentration >30 mg/dL 5. Neurological dysfunction - seizures or encephalopathy Table credits: Dr. Ramansh Bandhu Gupta

Criteria	City of Hope 2013 (COH) [23]	BMT-CTN2005 [25]	IVVG 2007 [26]	TA-TMA2014 (Jodele et al., 2015) [27]	Overall TMA 2014 (Cho et al., 2010) [28]	TA TMA 2014 (Uderzo et al., 2014) [29]	TA TMA 2019 (Li et al., 2019) [30]
Requirements for diagnosis	4 criteria - definite; 3 criteria - probable	All features should be present	All features should be present	>4/7 features at two time points in 14 days or renal biopsy showing TA-TMN	All features present at two time points	All features present	All features within 24 hrs, overall TA-TMN microangiopathy, definte MAHA plus organ dysfunction
Anemia or need for RBC transfusion	+	+	+	+	+	+	+
Thrombocytopenia/increased platelet transfusion needs	+	+	+	+	+	+	+
Increased LDH	+	+	+	+	+	+	+
Hemolysis (schistocytes)	+ (Present or nucleated RBC)	+ >2/HPF	+ >4% or 8/HPF	+	+ >2/HPF	+ >1-2/HPF	+ >2/HPF
Negative Coombs test (direct and indirect)	-	+	-	-	+	+	+
Renal dysfunction	+	+	-	+	-	+	+
Decreased haptoglobin	-	-	+	+	+	-	-
Normal coagulation profile	-	-	-	-	-	-	+
Elevated sC5b-9	-	-	-	+	-	+	-
Hypertension	-	-	-	+	-	+	-
Proteinuria	-	-	-	+	-	+	-
Renal biopsy	-	-	-	+not required but if done sufficient for diagnosis of TMA	-	-	
Neurological dysfunction	-	+	-	+	-	+	+

The fundamental distinction between immune-mediated and dose-dependent TMA resides in their underlying mechanisms and clinical manifestations. Immune-mediated TMA is characterized by an immune response directed against a specific drug, culminating in acute clinical features, and, notably, its occurrence is not correlated with trough drug levels. Conversely, dose-dependent TMA stems from the direct toxic effects of a drug on the microvasculature, resulting in a more gradual onset, and there is a potential link to drug trough levels, particularly evident in medications such as tacrolimus [15,24]. Furthermore, the combined use of tacrolimus and mTOR inhibitors can exacerbate the risk of DITMA. Sirolimus is more commonly associated with this complication than everolimus. The sequence of endothelial damage by CNIs, followed by mTOR inhibitors hindering endothelial repair, predisposes patients to post-transplant TMA. Monitoring the blood levels of both drugs may help prevent this complication [31].

**Figure 1:**
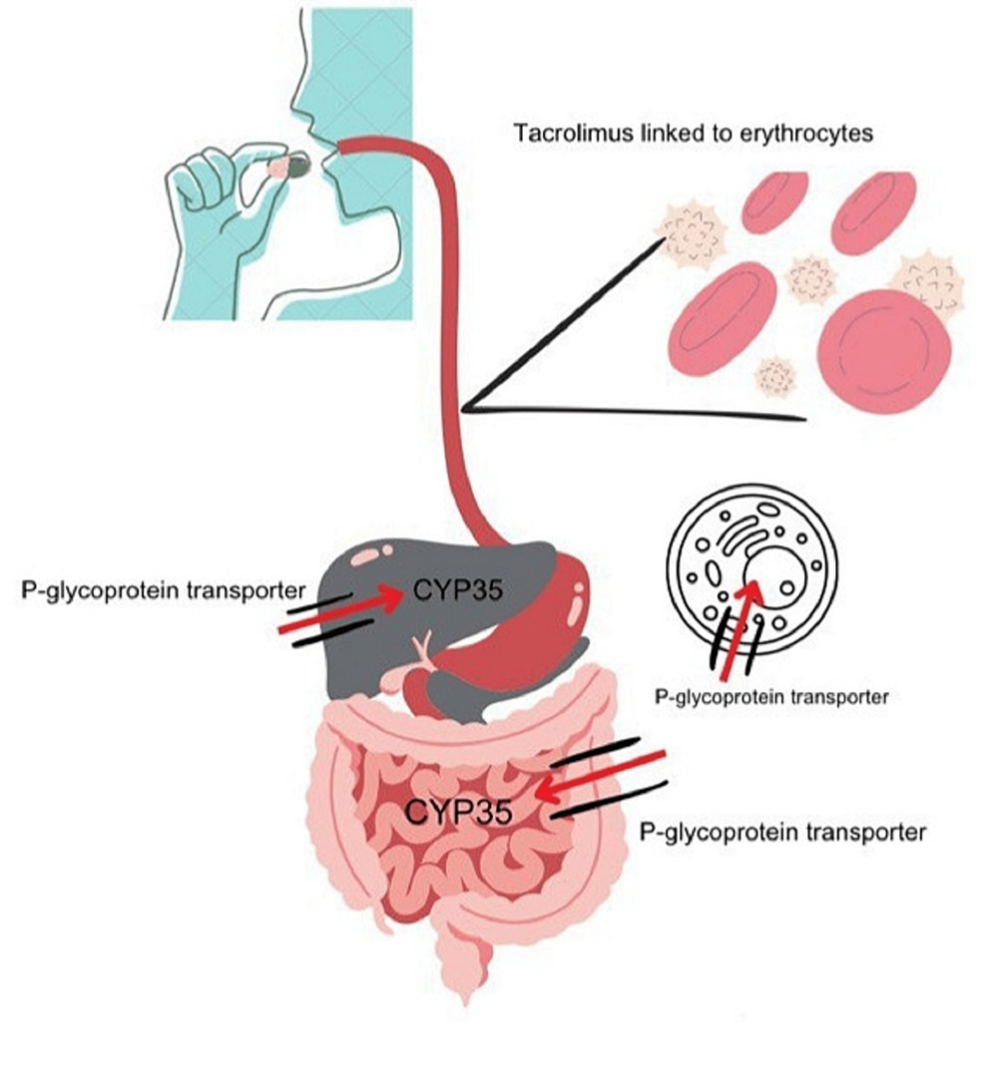
Oral metabolism of tacrolimus Image credits: Dr. Srinija Polishetti

**Figure 2:**
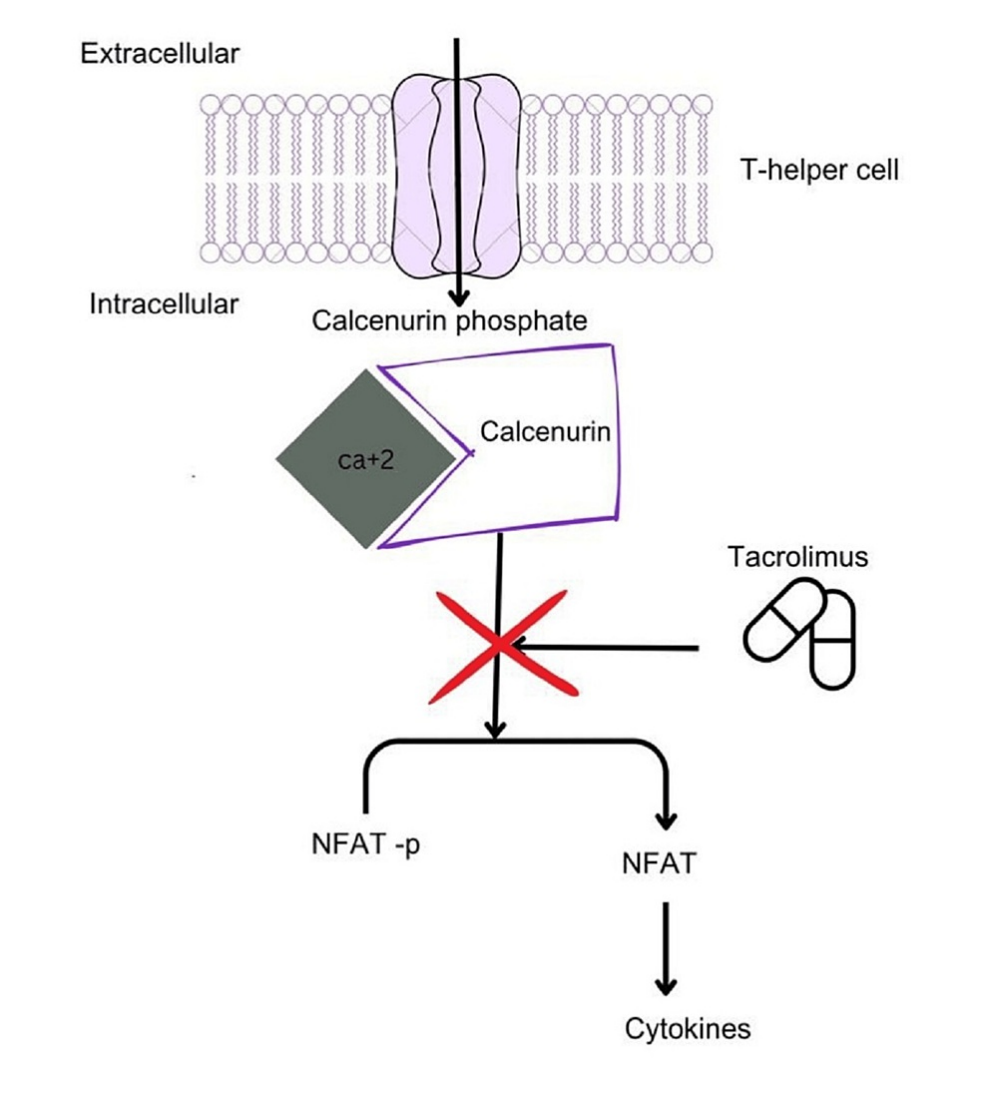
Mechanism of action of tacrolimus as an immunosuppressant Image credits: Dr. Srinija Polishetti

**Figure 3:**
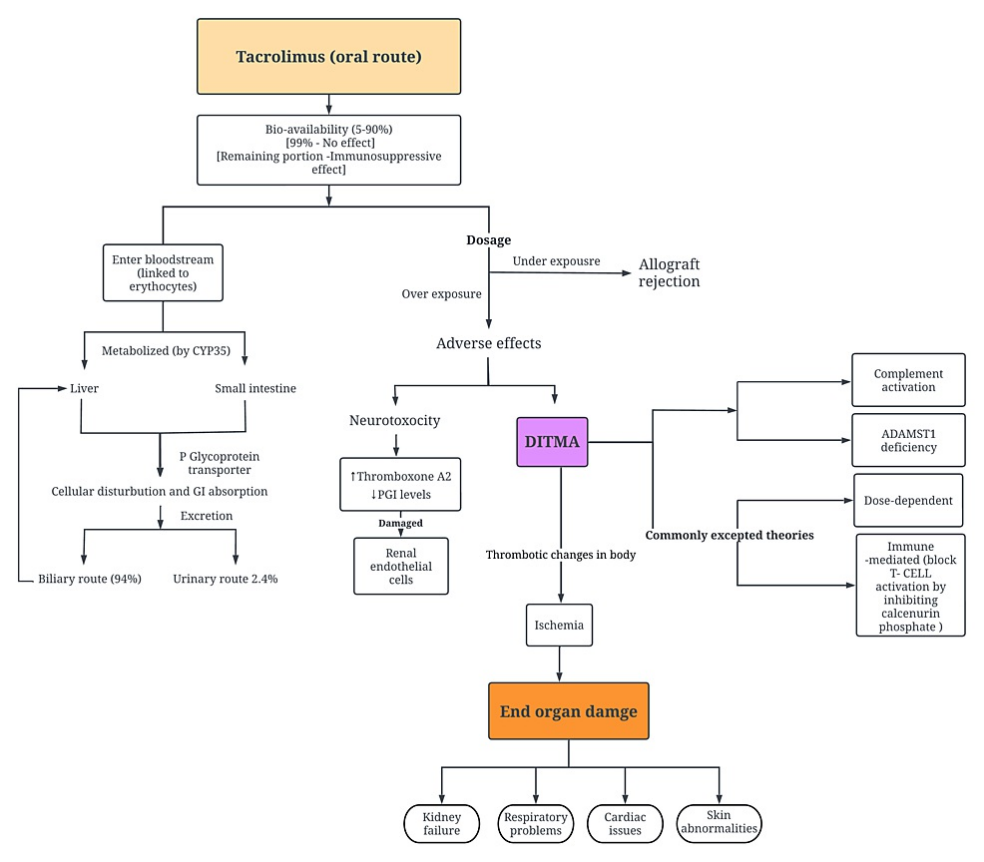
Flowchart depicting pathophysiology of how tacrolimus induces TMA Abbreviations - Cytochrome 450 P 3A5 (CYP35), A disintegrin and metalloproteinase with a thrombospondin Type 1 motif, member 13/von Willebrand's factor-cleaving protease (VWFCP)/(ADAMTS13), drug-induced thrombotic microangiopathy (DITMA), tacrolimus (TAC), thrombotic microangiopathy (TMA), nuclear factor of activated T-cells (NFAT), tecombinant NFAT1 (NFAT-P) Image credits: Dr. Srinija Polishetti

In conclusion, the pathophysiology of tacrolimus in transplant patients is a delicate balance between its potent immunosuppressive effects and the risk of adverse outcomes. The drug's variable half-life and complex elimination pathways necessitate careful management to avoid overexposure or underexposure. Figures 1-3 describe the metabolism and pathophysiology of TMA.

Discussion 

TMA is defined by a series of events that include endothelial cell damage, intravascular platelet activation, platelet-fibrin thrombi development, microcirculation restriction, and hemolytic anemia with platelet and erythrocyte consumption [32]. It can have a genetic cause (ADAMTS-13 deficiency and coagulation pathway mutations), as well as bacterial and viral infections, malignancy, pregnancy, autoimmune diseases, HIV, and some medications such as CNIs, bevacizumab, mitomycin C, and others [24,33]. Furthermore, females have a higher incidence of TMA in non-transplantation and transplantation settings [33].

Tacrolimus complexes with calcineurin-dependent proteins suppress cellular immunity and interact with the intracellular protein FKBP-12 [34]. TMA is an uncommon yet severe tacrolimus-related complication in 1-4.7% of adult transplant patients [35]. A survey of 91 solid organ transplant recipients with tacrolimus-associated TMA found that kidney transplant recipients accounted for 81% of cases, liver transplant recipients accounted for 8%, and lung and heart transplant recipients accounted for less than 1% [34]. Skin involvement has not traditionally been reported in DITMA cases, in contrast to thrombotic thrombocytopenic purpura and atypical hemolytic uremic syndrome [15]. Although there is no known mechanism of tacrolimus-associated TMA, endothelial injury has been linked to direct cytotoxicity, injury caused by platelet activation, increased von Willebrand factor (VWF), thrombomodulin, nitrous oxide, and prostacyclin production [34]. Patients with many prior treatments, such as those receiving a second hematopoietic cell transplant (HCT), appear at a higher risk of TMA when CNI and sirolimus are combined [36,23]. The clinical presentation of TMA is determined by the severity of organ vascular involvement, which might vary. Localized TMA patients typically exhibit graft dysfunction that worsens over time. Patients with systemic TMA exhibit clinical and biochemical HUS/TTP features [37]. The proposed diagnostic criteria for TMA display significant heterogeneity, mostly based on retrospective analyses. Initially, two consensus criteria were formulated for tacrolimus-induced TMA, namely, the bone marrow transplant clinical trials network (CTN-TMA, 2005) and the International Working Group of the European Group for Blood and Bone Marrow Transplantation (IWG-TMA, 2007) [38]. Cho et al. introduced an overall TMA (O-TMA) diagnostic criterion [28]. Subsequently, the Joint Study Group and City of Hope proposed two additional sets. DITMA may be suspected in any individual with an unexplained decrease in platelet count and schistocytes on the blood smear, typically with worsening kidney function. The systemic nature of the pathophysiology of TA-TMA can lead to renal insufficiency and failure, refractory hypertension, posterior reversible encephalopathy syndrome, seizures, altered mental status, pulmonary hypertension, diffuse alveolar hemorrhage, abdominal pain, GI ischemia and bleeding, and serositis, including pericardial and pleural effusions [39]. Given the high mortality rate, the presence of anemia, thrombocytopenia, and the presence of schistocytes on a peripheral blood smear is enough to warrant treatment. Other laboratory markers include decreased haptoglobin, high LDH, raised reticulocytes, and unconjugated hyperbilirubinemia [24]. Clotting profiles are abnormal in more than 30% of the patients, and extensive infectious work-up is mandatory, with a special focus on CMV, EBV, parvovirus B19, and HCV [32]. Classically, in the case of primary TMA, PLEX should be initiated right away, and the causative medication should be reduced or stopped or replaced with less nephrotoxic immunosuppressants such as sirolimus or mycophenolate mofetil [40,14]. If PLEX is not immediately available, plasma infusion therapy can be started [24]. Three incidences of tacrolimus-associated TMA were discovered by Russ et al. [41] in 31 recipients with high target trough levels of tacrolimus (10-15 ng/mL) and 35 recipients with low target trough levels of tacrolimus (3-7 ng/mL). Although it is debatable if lowering blood levels of TAC helps treat TAC-associated TMA, instances did improve when the target TAC trough level was dropped from 15 to 6-8 ng/mL. Humar et al. [42] also reported that TAC-associated TMA was improved only by a dose reduction of TAC. When a patient is not responding to PLEX and has normal ADAMTS-13 activity, eculizumab could be considered [24]. Although it has not been documented as a specific treatment for CNI-associated TMA, eculizumab, in the context of CNI-induced TMA, represents directed anti-complement therapy with a more immediate cessation of endothelial cell harm [43]. Furthermore, belatacept and eculizumab combination therapy offers a new therapeutic option for calcineurin-induced TMA and has potential benefits over conventional therapeutic protocols [43]. The cumulative incidence of response to treatment, including PE, was 60% and required control of coexisting graft-versus-host disease (GVHD) and infections. Even among responders, mortality remains high [36]. Nonetheless, TMA is a potentially fatal disorder. Despite comprehensive treatment, numerous fatal results have been described previously, necessitating early detection, aggressive treatment, and follow-up due to the danger of relapse [32].

## Conclusions

TMA is a significant organ transplantation complication caused mainly by the prothrombotic impact of CNIs. Tacrolimus, a highly effective immunosuppressive drug used in organ transplantation, lowers cellular immunity by forming complexes with calcineurin-dependent proteins and interacting with the intracellular protein. An endothelial injury marks tacrolimus-associated TMA. Although the mechanism of direct endothelial injury is not fully understood, it is thought to be immune-mediated or dose-dependent direct toxicity. The proposed diagnostic criteria for TMA display significant heterogeneity. There should be a high level of suspicion for the diagnosis of TA-TMA when patients present with an unexplained decrease in platelet count and schistocytes on the blood smear and elevated LDH, typically with worsening kidney function. Plasmapheresis and tacrolimus discontinuation are conventional treatment cornerstones for the primary TA-TMA. Newer biological medicines, such as eculizumab, a monoclonal antibody against complement component C5, and belatacept, a CTLA-4 inhibitor, have provided treatment alternatives for tacrolimus-induced TMA. Considering that TMA is a potentially lethal condition with frequent fatal outcomes despite comprehensive therapy, standardized, objective, and organ-specific criteria must be established to assist the early identification of TMA and its practical use in future clinical studies.
